# Research on Auxiliary Decision-Making System for Manned Underwater Vehicle Damage Management Based on Deep Reinforcement Learning

**DOI:** 10.3390/s26123678

**Published:** 2026-06-09

**Authors:** Qingchao Xu, Hui Feng, Haixiang Xu, Fang Tang, Yong Wang, Yifeng Chen, Liping Zhou

**Affiliations:** 1 Key Laboratory of High Performance Ship Technology (Wuhan University of Technology), Ministry of Education, Wuhan 430063, China; hdyywxchaos@whut.edu.cn (Q.X.);; 2 School of Naval Architecture, Ocean and Energy Power Engineering, Wuhan University of Technology, Wuhan 430063, China; 3 Wuhan Second Ship Design and Research Institute, Wuhan 430063, China

**Keywords:** underwater safety, damage management, decision support, cabin flooding, deep reinforcement learning, deep Q-network

## Abstract

In underwater navigation, MUVs risk damage from obstacles and equipment. Effective damage management supports timely decisions and maximizes functionality recovery. Existing approaches can be roughly categorized into rule-based reasoning, case-based reasoning and expert systems. However, the primary limitation of the existing approaches is their inability to adapt to dynamically changing scenarios. In this paper, an auxiliary decision-making system (ADMS) for manned underwater vehicle (MUV) damage management based on deep reinforcement learning (DRL) is proposed to address the problem of cabin flooding. This system is designed to provide auxiliary decision-making in emergency situations and help preserve MUV vitality. Furthermore, a comprehensive States–Actions cluster encompassing various damage management measures for real damage scenarios is constructed and digitized. Moreover, several novel reward functions are developed to ensure the DRL model obtains a safe strategy with ADMS operations. Finally, the MUV buoyancy and stability vitality evaluation criteria are defined and analyzed. The simulation results show that the auxiliary decision-making measures given by the ADMS in the damage state are effective and rational. The evaluation criterion for buoyancy vitality can exceed 38%, while the criterion for stability vitality can surpass 92%, with an optimal value exceeding 99%.

## 1. Introduction

With the continuous development and application of advanced underwater technologies, the threats faced by MUVs during underwater operations have become more diverse and unpredictable. Throughout the navigation process, MUVs may be struck and damaged by underwater obstacles or other equipment at any time, making rescue efforts in sudden and complex situations critical and urgent. For an MUV, if timely and effective rescue measures are not implemented after damage and flooding, its overall vitality could face a fatal risk. Therefore, a decision support system for damage management in MUV flooding is essential [1].

The research on the auxiliary decision-making system for MUV damage management in a broader sense is actually a vast and complex problem; it may involve state perception [2], situation prediction [3], auxiliary decision-making [4], and vitality assessment [5]. In addition, it is worth noting that MUVs may encounter multiple or even more complex constraints in real scenarios. This paper focuses solely on decision support in the damage state of cabin flooding, aiming to provide effective auxiliary measures for this specific situation.

Although existing methods for constructing decision support systems for damage management have been widely applied, such as rule-based reasoning [6], case-based reasoning [7], and expert systems [8], the primary limitation of the existing methods is their inability to adapt to the dynamic changes in MUV damage scenarios. Rule-based reasoning can only handle known situations due to its inflexible reasoning process. Case-based reasoning struggles to find suitable comparisons because of the numerous variables involved. Additionally, the knowledge base of expert systems often fails to fully capture the completeness of a given scenario. Specifically, the rules and knowledge representations used in traditional methods during the actual damage management process fail to encompass all possible scenarios. A method based on deep reinforcement learning (DRL) can significantly address this issue to a certain extent. Reinforcement learning (RL) enables agents to interact with the environment dynamically and receive feedback, making it more suitable for real-world scenarios; deep learning (DL) enhances the accuracy and reliability of the RL process. Therefore, DRL demonstrates better adaptability to dynamic and complex scenes compared to traditional methods.

In view of the research gap and challenges concerning MUV damage management, this paper proposes an auxiliary decision-making system (ADMS) for manned underwater vehicle (MUV) damage management based on deep reinforcement learning (DRL) to address the problem of cabin flooding. At first, a comprehensive States–Actions cluster of various damage management measures (including its digitization, which makes it easy to compile) is constructed to closely resemble a real MUV damage scenario. Moreover, according to the vital parameters of the MUV’s vitality and the measures of the auxiliary decision-making for damage management, several reward functions are designed to provide the DRL model with feedback on the MUV’s safety strategy during interactions with the environment. Furthermore, appropriate evaluation criteria are essential to assess the rationality and effectiveness of the designed DRL model. In this paper, two vital parameters related to the vitality of the MUV are selected as the design basis. Combined with the relevant MUV safety standards, the evaluation criteria that can represent the performance of the ADMS for MUV damage management based on DRL are designed and analyzed.

The main contributions of this study are summarized as follows.

- An auxiliary decision-making system (ADMS) for manned underwater vehicle (MUV) damage management based on deep reinforcement learning (DRL) is proposed, which is able to adapt to dynamically changing scenarios by interacting with the environment.
- A complete States–Actions cluster of various damage management measures in real MUV damage scenarios is constructed and digitized.
- Several novel reward functions are developed to ensure the DRL model obtains a safe strategy with ADMS operations.
- The MUV buoyancy and stability vitality evaluation criteria are defined and analyzed, which are utilized to assess the rationality and effectiveness of the ADMS.

The remainder of this paper is organized as follows. Section 2 reviews the relevant literature. In Section 3, the framework of the ADMS is introduced. Section 3 presents the construction of the damage States–Actions cluster, the design of the state–action value function based on function approximation, and the design of the reward functions. Simulation results and evaluation criteria are presented in Section 4. Finally, the conclusions of this study and future works are discussed in Section 5.

## 2. Literature Review

There are few studies on the auxiliary decision-making system of damage management for MUVs, but the research on damage control of surface ships is worth learning.

### 2.1. Damage Control System

Calabrese [9] developed a crucial application in the naval field called the damage control system (DCS), which is an information-retrieval and equipment-control system that allows ship personnel to detect, analyze, and handle various types of situations that endanger the safety of ships. Calabrese [10] described the development of a knowledge-based decision support system (KDSS) integrated within a DCS designed for a national navy. The KDSS uses a hybrid design and runtime knowledge model to assist damage control operators through a kill card function that supports damage identification, action scheduling and system reconfiguration. Later, some scholars made a series of improvements on the basis of this DCS system.

Hu [11] presented an M-H method-based decision support system (MHDSS) that could evaluate survivability and aid real-time decision-making during shipboard flooding accidents. It relies on a certain reasoning logic to select available counter-flooding tank (CFT) combinations to provide swift and effective decision-making support for shipboard personnel on warships. Li [12] constructed an intelligent decision support system for the damage control of damaged ships combined with data mining. Park [13] suggested a development procedure of damage control training scenarios using their survivability analysis results as a new concept of damage control training programs employing advanced systems such as a damage control console, an automation system, and kill cards. Kim [14] considered a crew location recognition system for onboard incident response combining radio-frequency identification (RFID) and micro-electro-mechanical systems (MEMSs) for application to the existing computerized incident response system or damage control system (DCS) adopted by large passenger ships and naval vessels. Braidotti [1] developed effective onboard decision support systems (DSSs) capable of objectively assessing and measuring the overall ship safety during navigation as well as during an emergency. In the system, a fuzzy analytical hierarchical process is applied to define the weights of importance of a large number of criteria and sub-criteria composing a risk-based framework.

### 2.2. Decision-Making for Damage Management

Most of the existing methods are divided into three categories, including rule-based reasoning, case-based reasoning and expert system. Iqbal [15] presented a rule-based decision-making method for the maritime rescue stage. The rules are derived from the implicit knowledge of domain experts, thus facilitating the implementation of rule-based decision support system models. Chang [6] developed an approach to evaluate the risk level of major hazards associated with Maritime Autonomous Surface Ships (MASSs). To this extent, a Failure Modes and Effects Analysis (FMEA) method was used in conjunction with Evidential Reasoning (ER) and Rule-based Bayesian Network (RBN) to quantify the risk levels of the identified hazards. Zhao [16] proposed a method of extracting and constructing naval surface vessel operation decision-making rules based on scenario analysis. The template specifications of the Event Condition Action (ECA) rules were defined, and a consistency detection method of ECA rules based on SWRL was proposed. However, rule-based reasoning is constrained to familiar situations due to its rigid logical framework and cannot adapt to new scenarios.

Storvik [17] created a decision support system for helping search-and-rescue operators make quick decisions based on the situation assessment. The main objective of this research was to design and develop the retrieval process in the case-based reasoning (CBR) component for predicting the hypothesis of the best action given a goal and a situation assessment. Zhu [7] designed a framework case-based decision-making reasoning method that integrates multiple pieces of information. This method is applied to the auxiliary decision-making of MUV damage management, and an auxiliary decision-making model suitable for MUV flooding emergency is constructed. Louvros [18] presented a novel approach combining machine learning (ML) and case-based reasoning (CBR) that serves the purpose of a fast decision support tool for ship damage stability that is also able to provide information regarding the ongoing casualties utilizing prior knowledge gained from simulations. However, case-based reasoning encounters difficulties in identifying appropriate comparisons because of the complexity and variety of influencing factors.

Li [8] proposed a hybrid emergency decision-making approach based on the fuzzy expert system to ensure much more effective responses in realistic cases. The proposed hybrid approach assists decision-makers in dealing decisively with the ambiguity associated with the data for assessing and evaluating emergency circumstances. Lee [19] developed a performance-based on-board damage control system for ships instituted when a fire or flooding occurs. Their system can be used as an evaluative tool for accident-response crew training. Bolbot [20] proposed a novel hybrid, semi-structured process for hazardous scenario identification and ranking by evaluating a number of experts participating in a series of sessions. This method integrates operational and functional hazard identification approaches whilst considering safety, security and cybersecurity hazards. However, expert systems often fail to comprehensively represent all aspects of a given scenario due to the limitations of their knowledge base.

### 2.3. Deep Reinforcement Learning

Deep reinforcement learning (DRL) is a machine learning (ML) method that combines deep learning (DL) and reinforcement learning (RL). It uses deep neural networks to approximate strategies or value functions to solve high-dimensional and complex dynamic decision-making problems. Due to its low complexity, it is preferred to use the model-free DRL method in the auxiliary decision-making of damage management. The model-free DRL method can usually be divided into value function-based DRL and policy gradient-based DRL [21]. The value-based DRL is applicable to discrete action spaces. The typical value-based DRL algorithm is deep Q-learning (DQN), including improved algorithms of DQN like double DQN (DDQN). Li [22] proposed an improved DQN algorithm to provide decision-making for ship collision avoidance that employs the APF method to optimize the action space and rewards of DQN. Mohammadi [23] developed a DDQN-based approach to optimize maintenance and renewal planning. This approach optimizes renewal and maintenance planning over the railway planning horizon by considering cost-effectiveness and risk reduction. Policy gradient-based DRL methods, including the Deep Deterministic Policy Gradient (DDPG) method, the Proximal Policy Optimization (PPO) method, and so on, are good at dealing with a continuous action space. Yao [24] proposed a Double Critic Network (DCN-DDPG) algorithm in the path-tracking controller of an unmanned vehicle to autonomously learn the path-tracking capability of the vehicle by interacting with the CARLA environment. Qi [25] developed a scheduling strategy optimization method using the Proximal Policy Optimization (PPO) algorithm. This method enables intelligent pilots to automatically learn and adjust scheduling strategies to adapt to complex rescue environments and varying task demands. This paper utilizes the DQN algorithm, which is suitable for environments with limited discrete action spaces. DQN exhibits good convergence and stability, along with low computational complexity, thus meeting the design requirements of MUV damage management.

## 3. The Proposed Method

### 3.1. Framework

**Figure 1:**
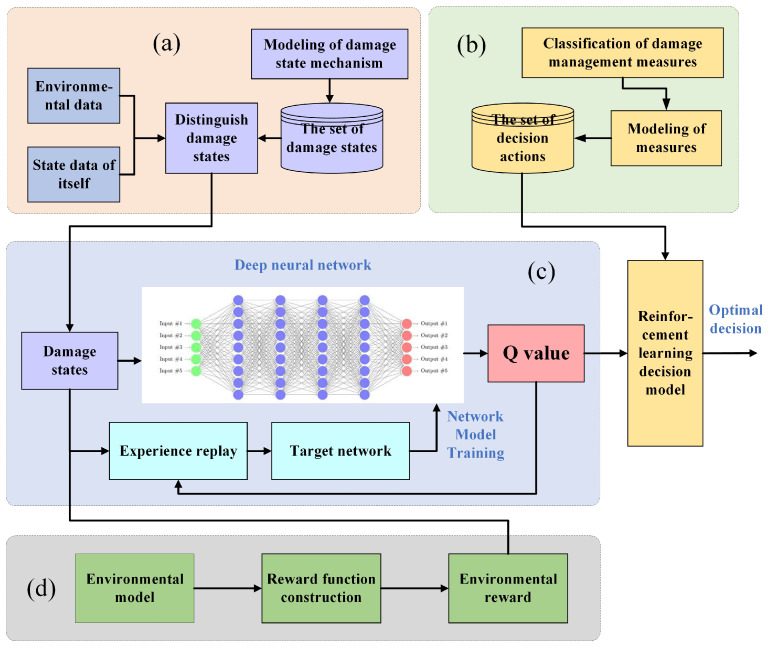
The framework of the proposed ADMS based on DRL. (**a**) Construction of damage state set. (**b**) Construction of decision-making action set. (**c**) Design of state–action value function. (**d**) Design of reward functions.

The framework of the ADMS for damage management based on DRL (DQN) is illustrated in Figure 1. First, establish the damage state set (Figure 1a) and the decision-making action set (Figure 1b). Next, design several reward functions and obtain the rewards (Figure 1d). Then, define the state–action value function (Figure 1c) and use a deep neural network to approximate the Q-value. Finally, derive the optimal decision as the output. The above process can be summarized as follows: 1. Construct a set of states and actions (states and actions). 2. Design a reward function (reward). 3. Design a value function and train the neural network (agent). 4. The agent interacts with the environment model (environment). This process satisfies the five key elements of reinforcement learning: agent, environment, state, action, and reward. In addition, the environmental model (dynamics model) selected in this paper is basically consistent with the systems recommended by the International Towing Tank Conference (ITTC) and the Society of Marine Engineering (SNAME) [26].

### 3.2. Information Processing and Construction of Damage States–Actions Cluster

**Figure 2:**
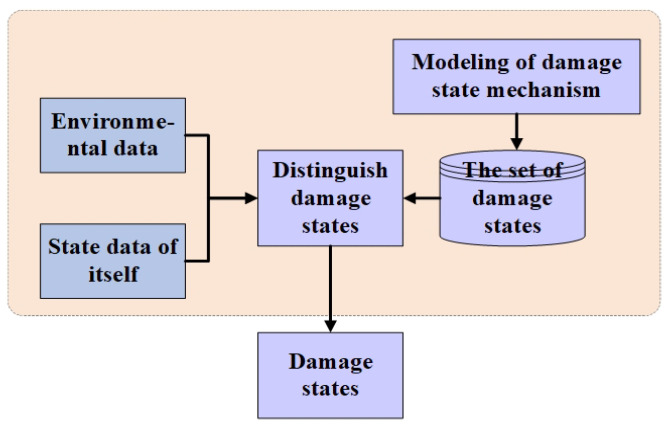
Flow chart of damage state construction (Figure 1a).

The state is a fundamental factor in the reinforcement learning (RL) model, serving as the basis for addressing a range of decision-making problems. Therefore, the definition of the damage management state within the ADMS module for damage management is critical for effectively applying RL methods in auxiliary decision-making. The damage mechanism of the MUV’s cabin is analyzed and its damage state is modeled to construct a comprehensive set of potential damage states. The damage states are distinguished by the existing environmental data and its own state data. The specific process is illustrated in Figure 2. The damage mechanism is analyzed and its damage state is modeled to construct a set of damage states; the damage state is determined by using existing environmental data and its own state data.

**Figure 3:**
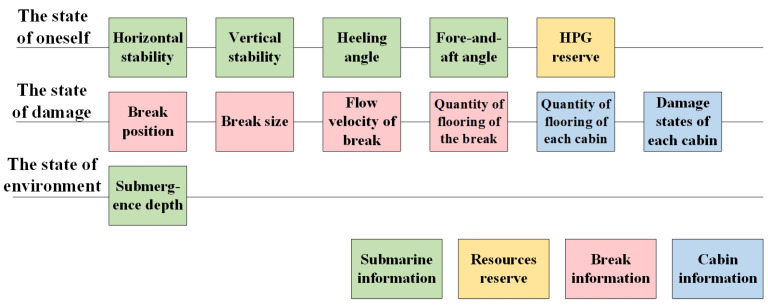
State composition of auxiliary decision-making model.

The damage state of the auxiliary decision-making model based on RL mainly consists of three parts: its own state, damage state and environmental state. Its own state includes the MUV’s own stability, posture (marked in green) and damage management resource reserve (marked in yellow); the damage state includes the break information (marked in pink) and the cabin information (marked in blue); and the environment state includes static environment information and dynamic environment information (this paper only considers the submergence depth where the MUV is located in the direction of heaving). The specific state factors are illustrated in Figure 3, where high-pressure gas is abbreviated as HPG on the right side of the first line.

**Figure 4:**
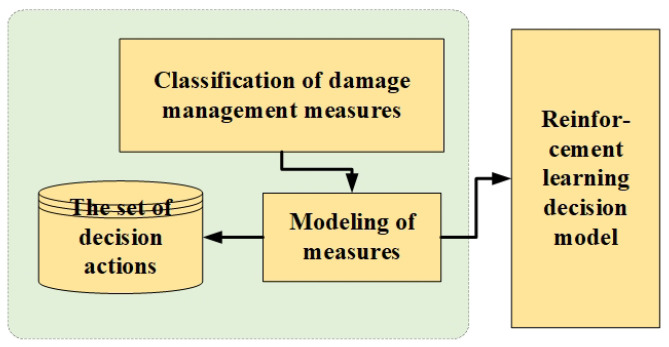
Process of disposal measures (Figure 1b).

Action represents the output of the RL model, and the refinement of the RL action output is one of the key issues of the auxiliary decision model. The action output of the ADMS based on DRL is the damage management measure, and its process is illustrated in Figure 4. The steps of the damage management measures are as follows: 1. Classifying the damage management measures according to the damage management manual. 2. Creating digital models for the various measures. 3. Constructing an intelligent decision–action set.

**Figure 5:**
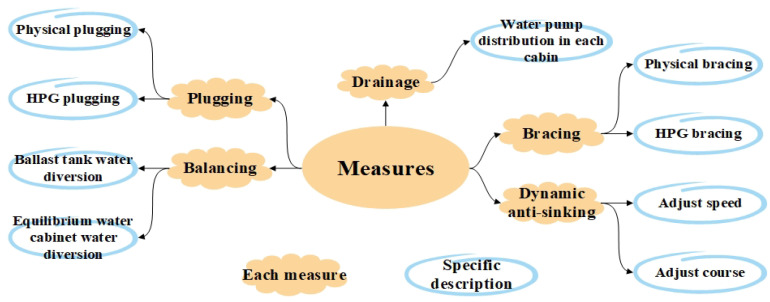
Disposal measures.

According to the related damage management manual [27,28], the damage management measures are divided into five categories: ‘plugging’, ‘bracing’, ‘drainage’, ‘balancing’ and ‘dynamic anti-sinking’. These are specifically described as blocking the water inlet break (plugging), closing the cabin and supporting the roof bulkhead (bracing), removing the water in the cabin (drainage) and balancing the posture of the damaged MUV (balancing). The specific description of the disposal measures is illustrated in Figure 5.

**Figure 6:**
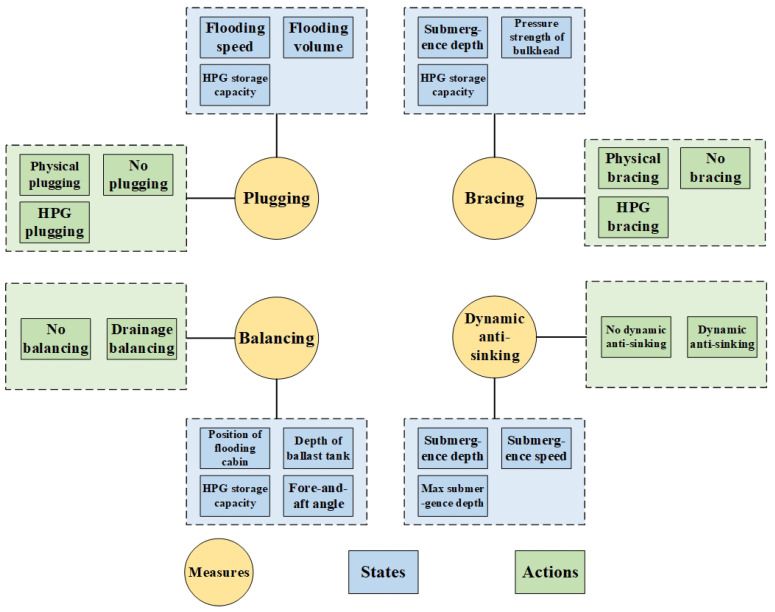
States–Actions cluster.

On the basis of the above auxiliary decision-making measures, this paper constructs the corresponding continuous state space and discrete action space for the measures of ‘plugging’, ‘bracing’, ‘balancing’ and ‘dynamic anti-sinking’. The mapping relationship between the two spaces is realized by designing the state–action value function. The specific States–Actions cluster of each damage management measure is illustrated in Figure 6.

‘Plugging’: ‘Physical plugging’ refers to the process of sealing the damaged area using physical equipment operated by personnel inside the cabin. The plugging effect is strengthened with the extension of time, but with the increase in the break size and the submergence depth, the physical plugging effect is greatly diminished. ‘HPG plugging’ refers to filling the damaged cabin with a large volume of HPG to ensure that the air pressure inside the cabin is greater than or equal to the water pressure outside the MUV, thereby achieving a plugging effect. The time required for this process can often be considered negligible. Nonetheless, as the submergence depth increases, the rising water pressure may slightly reduce the effectiveness of this plugging method.

‘Bracing’: ‘No bracing’ refers to the absence of any measures taken to support the adjacent cabins of the damaged cabin. ‘Physical bracing’ refers to the implementation of appropriate physical protection measures to enhance the structural strength, thereby improving the MUV’s resistance to external shocks. ‘HPG bracing’ refers to filling a significant volume of HPG into the adjacent cabins of the damaged cabin, ensuring that the pressure difference between the adjacent cabin and the damaged cabin remains less than or equal to the compressive strength of the bulkhead.

‘Balancing’: ‘Drainage balancing’ refers to the discharge of ballast water through HPG to increase buoyancy, slow down the diving speed, and balance the pitch moment. (Here, the drainage measures are integrated into the balancing measures and are characterized as an action).

‘Dynamic anti-sinking’: ‘Dynamic anti-sinking’ refers to the calculation of the corresponding forward or backward speed through the current MUV attitude and diving speed, thereby alleviating the submergence depth drop caused by damaged flooding.

**Figure 7:**
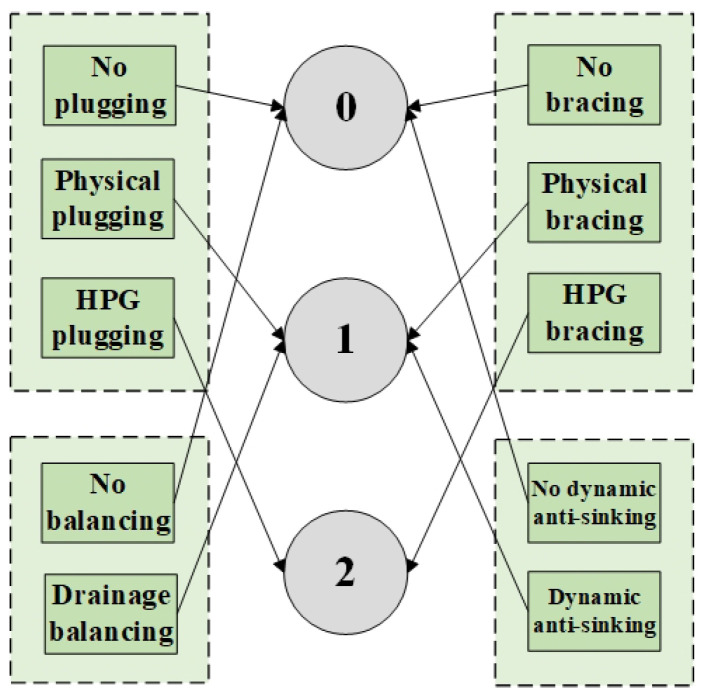
Action digitalization. (The numbers represent the corresponding actions in the algorithm).

In the process of implementing the algorithm, the corresponding digital processing of the action is needed, with the specific results shown in Figure 7.

As shown in Figure 7, four different measures are combined to obtain 36 schemes. The RL algorithm calculates the Q value of each scheme and selects the one with the highest Q value for output. In the algorithm design, each scheme is represented by a set of integer strings. For example, ‘1101’ denotes ‘physical plugging’, ‘physical bracing’, ‘no balancing’, and ‘dynamic anti-sinking’.

### 3.3. The Design of the State–Action Value Function

In the RL model, the value function is the basis for its strategy evaluation. In traditional RL approaches, such as Q-learning, the state–action value function is represented using a Q-value table, with its size determined by the system’s states [29,30]. However, constructing the state–action value function based on a table becomes challenging in complex continuous state spaces. In the actual damage management system, the damage scenario can be highly intricate, making it difficult to define discrete states, and even if they can be defined, the number may be prohibitively large. Consequently, in high-dimensional and continuous state spaces, the focus of intelligent auxiliary decision-making must be on developing effective and reasonable methods for constructing the value function.

Function approximation [31] involves fitting a function to the input–output relationship, and in the context of RL models, the value function represents the relationship between the action and the state with its corresponding value [32]. Consequently, constructing the value function through function approximation is an effective strategy for addressing high-dimensional and continuous state space inputs. Function approximation methods include techniques such as machine learning, artificial neural networks, and curve fitting. Recently, methods based on machine learning and artificial neural networks have emerged as research hotspots. The artificial neural network is one of the most significant methods of function fitting [33]. It not only has a convergence guarantee but also has an excellent fitting ability in complex high-dimensional environments. Therefore, utilizing neural networks to fit the state–action value function enhances the adaptability of RL algorithms in high-dimensional settings.

**Figure 8:**
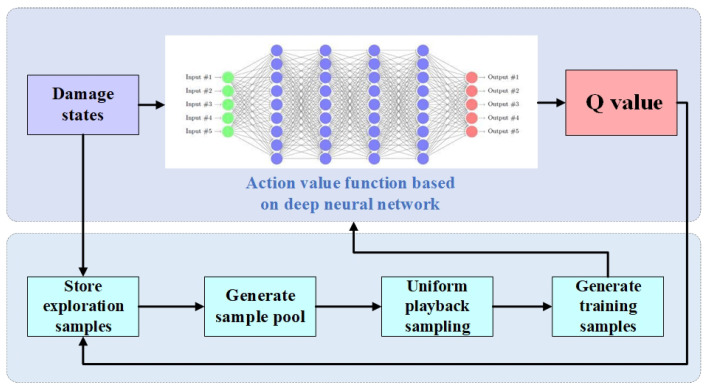
Neural network training sample generation process (Figure 1c).

**Figure 9:**
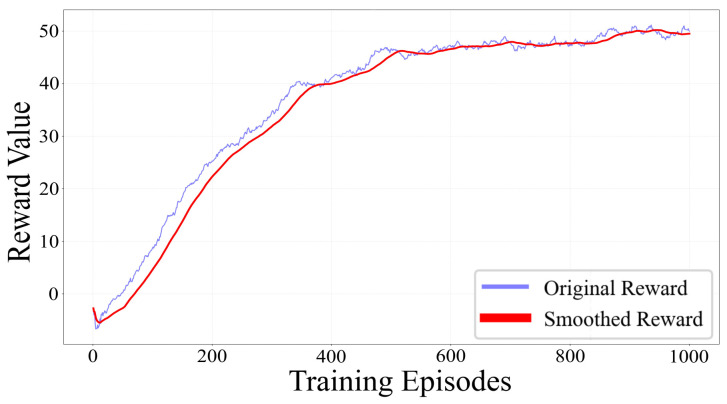
Training reward curve diagram.

This paper employs a deep neural network to construct the state–action value function of the RL model, and its principle in damage management decision-making is shown in the upper half of Figure 8. The input of the state–action value function based on the deep neural network is the damage state (the specific state factors are shown in Figure 3), and the output is the Q value of each damage-management action. The network is utilized as a nonlinear function approximator, enabling the calculation of Q values for each state–action pair, and then the action with the highest Q value is selected using a greedy strategy. The specific parameters of the deep neural network and algorithm are shown in Table 1. The neural network has a total of 4 layers (input layer + 2 hidden layers + output layer). A 4-layer network can extract features without making training difficult due to being too deep. The number of neurons in each layer gradually increases from the input layer to the output layer (7 → 12 → 24 → 48). The network extracts basic features in the front layer and performs higher-level decision-making in the back layer. The learning rate 0.01 is a commonly used conservative value in reinforcement learning that can ensure learning speed while avoiding training oscillations. The discount factor 0.9 indicates a greater emphasis on long-term returns. The Greedy parameter 0.9 represents a 90% probability of choosing the action currently considered optimal, and a 10% probability of random exploration. The training rounds are set to 1000, and the batch size is 128. The reward curve for reinforcement learning training is shown in Figure 9. As shown in Figure 9, the reward value tends to stabilize when the number of training rounds is around 500.

**Figure 10:**
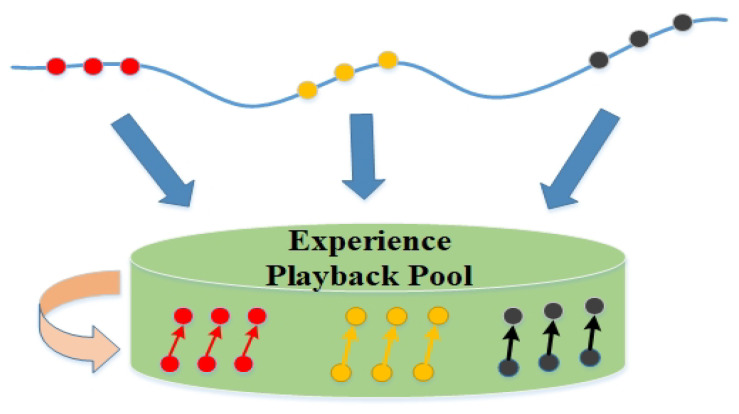
Experience playback mechanism principle. (Large arrows represent experience gathering, while small arrows represent the pairing of states with actions).

In this paper, after utilizing the neural network to construct the state–action value function, the empirical playback mechanism is employed to obtain the effective samples, and this principle is illustrated in Figure 10. Firstly, the initial model interacts with the environment to gather a series of samples, including states, actions, and rewards, which are stored in the experience pool. Subsequently, training samples are selected using a uniform sampling mechanism to train the neural network model. This process is shown in the lower half of Figure 8.

The commonly used parameter-update method is realized by gradient descent [34]. When the weight vector is updated by gradient descent in the RL based on function approximation, only the influence of changing the weight vector on the value function is considered, and the influence of change in the state–action on the value estimation is not considered. Therefore, in the parameter-update of the RL based on function approximation, only a part of the gradient is considered. This method is called semi-gradient parameter-update [35]. The objective of parameter-update is to minimize the mean square of the temporal difference error (TD Error). The loss function L(ω) of the Q network is constructed in the form of the mean square error (MSE) [36], and the loss function is minimized to find the optimal value of the parameter $\hat{\omega }$, as presented in Equations (1) and (2).

(1) $$\;\hat{\omega }=\arg\min_{\omega }L\left(\omega \right)$$

(2) $$\begin{array}{c} L\left(\omega \right)=\frac{1}{N}\sum_{i=1}^{N}\left[Q(S_{t},A_{t};\omega )\right.\\ {\left.-\left(R_{t+1}+\gamma \cdot max_{a}\hat{q}\left(s_{t+1},a_{t};\omega \right)\right)\right]}^{2} \end{array}$$

In Equation (2), L(ω) is the loss function and N is the number of samples. The calculation formula based on the semi-gradient parameter-updating method in this paper is presented in Equation (3).

(3) $$\;\omega \leftarrow \omega +\alpha \rho_{t}\left[G_{t}-\hat{q}\left(s_{t},a_{t};\omega \right)\right]\nabla \hat{q}\left(s_{t},a_{t};\omega \right)$$

In Equation (3), ω is the weight vector of the value function of the state–action, α is the learning rate, $\hat{q}\left(s_{t},a_{t};\omega \right)$ is the approximation value function of the state–action at the current time, $\nabla \hat{q}\left(s_{t},a_{t};\omega \right)$ is the partial derivative of the value function to the weight vector ω, $\rho_{t}$ is the sampling ratio at moment *t*, and $G_{t}$ is the return at moment t (goal), and the calculation method is presented in Equation (4).

(4) $$\;G_{t}=R_{t+1}+\gamma \cdot \hat{q}\left(s_{t+1},a_{t};\omega \right)$$

**Figure 11:**
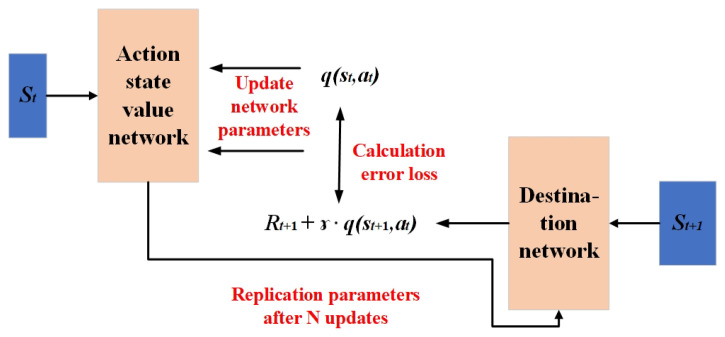
Update of network parameters based on target network mechanism.

In Equation (4), $R_{t+1}$ is the environmental reward at the moment t+1, and γ is the discount factor. The target network mechanism [37] is used to calculate $Q(S_{t},A_{t};\omega )$, then the parameters of the state–action value function based on the neural network are updated, as presented in Equation (5) and illustrated in Figure 11. In Figure 11, the output of the main network is compared with the target value to calculate the error loss, and this loss is used to update the parameters of the action state value network through backpropagation. After a certain number of N updates, the system copies the parameters of the action state value network to the target network to maintain the relative stability of the target network and avoid oscillations and divergences during training.

(5) $$\begin{array}{c} Q\left(S_{t},A_{t};\omega \right)\leftarrow Q\left(S_{t},A_{t};\omega \right)+\alpha [R_{t+1}+\\ \gamma \cdot max_{a}Q\left(s_{t+1},a_{t};\omega \right)-Q\left(S_{t},A_{t};\omega \right)] \end{array}$$

### 3.4. Reward Function Design

The main function of the reward function is to optimize the guiding measures. According to the vital parameters of the auxiliary decision-making for damage management, five reward functions are set up: the submergence depth reward function, the fore-and-aft angle reward function, the intake speed reward function, the HPG reward function and the dynamic anti-sinking reward function.

The specific formula for the reward function of the submergence depth is presented in Equations (6) and (7) as follows:

(6) $$\;R_{d}={(20}^{-\beta_{0}}-1)\cdot \alpha_{0}$$

(7) $$\;\beta_{0}=\frac{\left(D_{t}-D_{0}\right)}{D_{0}}\;$$

In Equation (6), $R_{d}$ is the reward value of the submergence depth and $\alpha_{0}$ is the reward expansion factor. In Equation (7), $\beta_{0}$ is the proportional factor, and $D_{t}$ and $D_{0}$ are the current submergence depth and the initial submergence depth.

The specific formula for the reward function of the fore-and-aft angle is presented in Equation (8), as follows:

(8) $$\;R_{a}=\left\{\begin{array}{cc} 0.2\cdot \alpha_{1} & \left|\theta \right|<9^{\circ }\\ 1.5^{-\left|\theta \right|}\cdot \alpha_{1} & 9^{\circ }<\left|\theta \right|<20^{\circ }\\ -\alpha_{1} & \mathrm{else} \end{array}\right.$$

In Equation (8), $R_{a}$ is the reward value of the fore-and-aft angle, $\alpha_{1}$ is the reward expansion factor, and θ is the fore-and-aft angle.

The specific formula for the reward function of the intake speed is presented in Equation (9), as follows:

(9) $$\;R_{v}={(10}^{-v_{t}}-1)\cdot \alpha_{2}$$

In Equation (9), $R_{v}$ is the reward value of the intake speed, $\alpha_{2}$ is the reward expansion factor, and $v_{t}$ is the intake speed.

The specific formula for the reward function of the HPG is presented in Equations (10) and (11), as follows:

(10) $$\;R_{g}={(10}^{-\beta_{1}}-1)\cdot \alpha_{3}$$

(11) $$\;\beta_{1}=1-\frac{\left({\mathrm{Vol}}_{\mathrm{gt}}-{\mathrm{Vol}}_{g0}\right)}{{\mathrm{Vol}}_{g0}}$$

In Equation (10), $R_{g}$ is the HPG reward value and $\alpha_{3}$ is the reward expansion factor. In Equation (11), $\beta_{1}$ is the proportional factor, and ${\mathrm{Vol}}_{\mathrm{gt}}$ and ${\mathrm{Vol}}_{g0}$ are the HPG storage at the current moment and the initial moment.

The specific formula for the reward function of dynamic anti-sinking is presented in Equation (12), as follows:

(12) $$\;R_{m}=\left\{\begin{array}{cc} -0.2\cdot \alpha_{4} & \mathrm{Dynamic}\;\mathrm{anti}\text{-}\mathrm{sinking}\\ 0 & \mathrm{No}\;\mathrm{dynamic}\;\mathrm{anti}\text{-}\mathrm{sinking} \end{array}\right.$$

In Equation (12), $R_{m}$ is the dynamic anti-sinking reward value and $\alpha_{4}$ is the reward expansion factor.

The complete algorithm steps for the ADMS damage management based on DRL (DQN) are as follows: Step 1. State-sensing (obtain the initial value of the current state). Step 2. Q-Value evaluation (neural network calculates the Q-value). Step 3. Action selection (ϵ-greedy strategy selects the optimal action). Step 4. Strategy execution (perform operations). Step 5. State transition (calculates the new state). Step 6. Reward calculation (comprehensive evaluation of decision-making effectiveness). Step 7. Weight update (TD learning updates network parameters). Step 8. Experience storage (store in memory for replay training). Step 9. Termination check (check if the termination condition has been met). Step 10. Loop iteration (return to step 1 to continue learning).

## 4. Simulation Analysis

### 4.1. Analysis of Influencing Factors

#### 4.1.1. Size of Break

The size of the break primarily affects the intake speed. In order to explore the specific impact of the size of the break on the auxiliary decision-making, three working conditions are designed, as shown in Table 2. The three working conditions are set up. Based on the actual cabin layout of a certain MUV, the environment is designed so that the MUV consists of six cabins, numbered 1–6 from bow to stern. If flooding occurs in the No. 2 cabin (the bow of the MUV), and the breach is assumed to be circular with a radius of 0.1/0.5/1.0 m, the initial submergence depth of the MUV is 10 m underwater, and the response time from cabin flooding to decision-making is 10 s.

**Figure 12:**
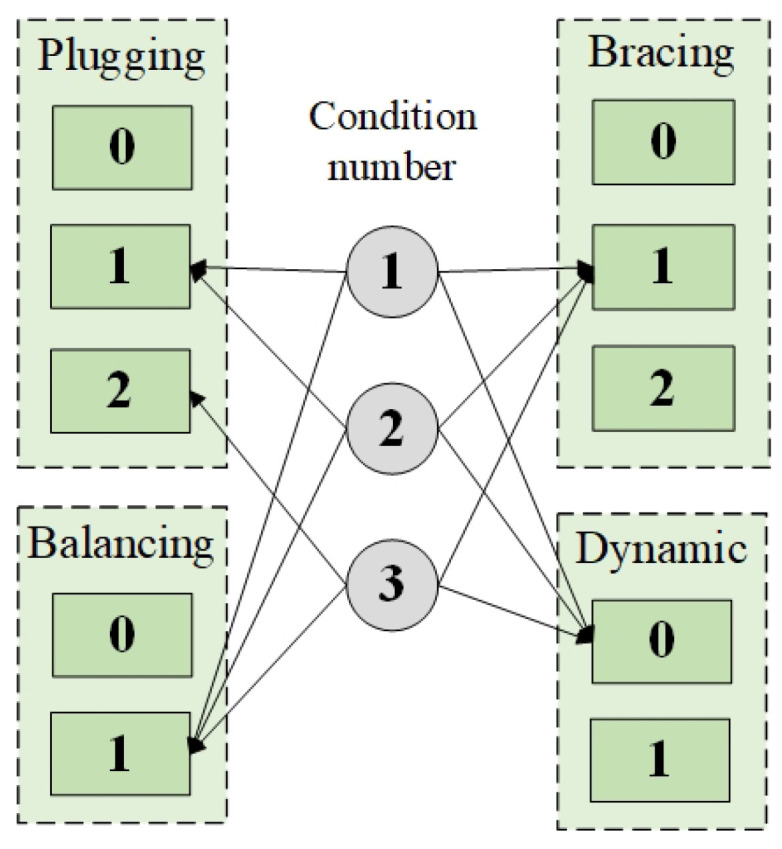
Auxiliary decision-making measures (Corresponding to Figure 7) under different working conditions (Table 2).

**Figure 13:**
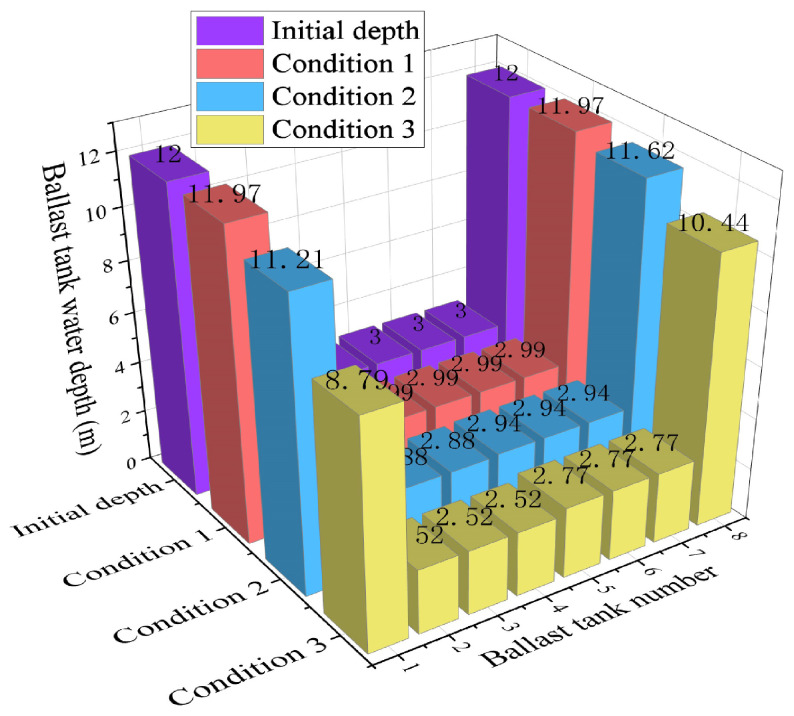
The variation in water depth for each ballast tank in different breaks.

**Figure 14:**
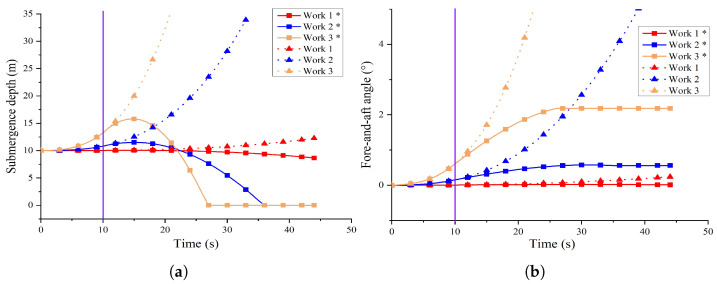
The state changes under different break sizes. (**a**) Submergence depth. (**b**) Fore-and-aft angle.

**Figure 15:**
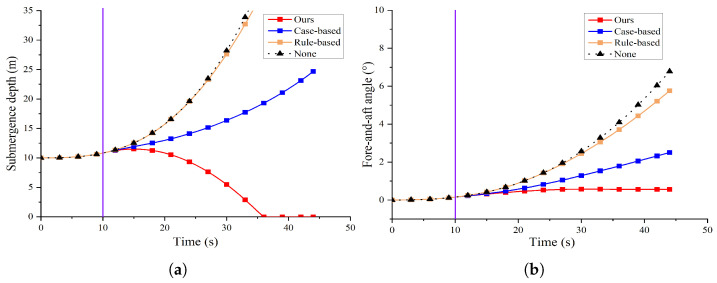
The state changes based on different methods (condition 2 from Table 2). (**a**) Submergence depth. (**b**) Fore-and-aft angle.

For the three working conditions with different break sizes, the strategy based on the ADMS is illustrated in Figure 12. Figure 13 shows the change in water depth for each ballast tank under different working conditions, while Table 3 presents the usage of HPG under different working conditions. The MUV state changes under different break sizes after auxiliary decision-making are illustrated in Figure 14a,b. The longitudinal purple line represents the auxiliary decision-making measures with a response time of 10 s. Having ‘*’ indicates a parameter change resulting from the implementation of an auxiliary decision, while no ‘*’ indicates a parameter change without an auxiliary decision. Taking condition 2 in Table 2 as an example, the state changes based on different methods are shown in Figure 15a,b.

As shown in Figure 13, taking the No. 1 ballast water tank as an example, the water depth of the ballast tank is 11.97 m in condition 1, 11.21 m in condition 2, and 8.79 m in condition 3. The lower the water depth, the more HPG is needed for purging. As shown in Figure 14a, work 3 and 2 surfaced in 27 s and 36 s, respectively, while work 1 floated slowly. Furthermore, compared to the case where no action was taken, the depth of all working conditions decreased to varying degrees after action was taken within 10 s. As shown in Figure 14b, after the measures were taken, work 3 stabilized at 2.2° in approximately 30 s, work 2 stabilized at 0.6° in approximately 30 s, and work 1 continued to approach 0°. Furthermore, the fore-and-aft angles under all three working conditions were reduced to varying degrees after the measures were taken. Therefore, the break size significantly influences the intake speed, which directly affects the inlet volume, consequently affecting the implementation of the measures, especially the demand for HPG. Variations in HPG usage lead to differences in the buoyancy and pitch moment during the MUV’s emergency response, ultimately impacting parameter changes. Generally, the larger the breach in the bow cabin, the greater the fore-and-aft angle. This also increases the demand for HPG, leading to MUV surfacing and thus a shallower submergence depth.

As shown in Figure 15a, the depth obtained using the method proposed in this paper reaches the surface in about 36 s, while the case-based method reaches 17 m and the reasoning-based method reaches more than 35 m. Moreover, the depth obtained using the latter two methods continues to decrease over time. As shown in Figure 15b, the angle obtained using the method proposed in this paper reaches 0.5° in about 36 s, while the case-based method reaches 1.8° and the reasoning-based method reaches 3.8°. Moreover, the angle obtained using the latter two methods continues to increase gradually over time. It also can be seen that the method proposed in this paper significantly improves upon reasoning-based and case-based methods in terms of both its depth and pitch angle results.

#### 4.1.2. Personnel Factor

Personnel factors primarily influence the implementation time of the measures, referred to as the response time. Response time is defined as the duration from making a decision to its complete implementation. When cabin personnel is sufficient, the response time corresponds to the basic response time, assumed to be 10 s in this paper. However, if personnel is insufficient, the response time increases beyond the basic value. To analyze the specific impact of human factors on auxiliary decision-making, the three working conditions are designed in Table 4. These three working conditions are also set up, and water also entered the No. 2 cabin (the bow of the MUV). The breach is assumed to be circular, with a radius of 0.5 m. The initial submergence depth of the MUV is 10 m underwater, and the response time from cabin flooding to decision-making is 10/15/20 s.

**Figure 16:**
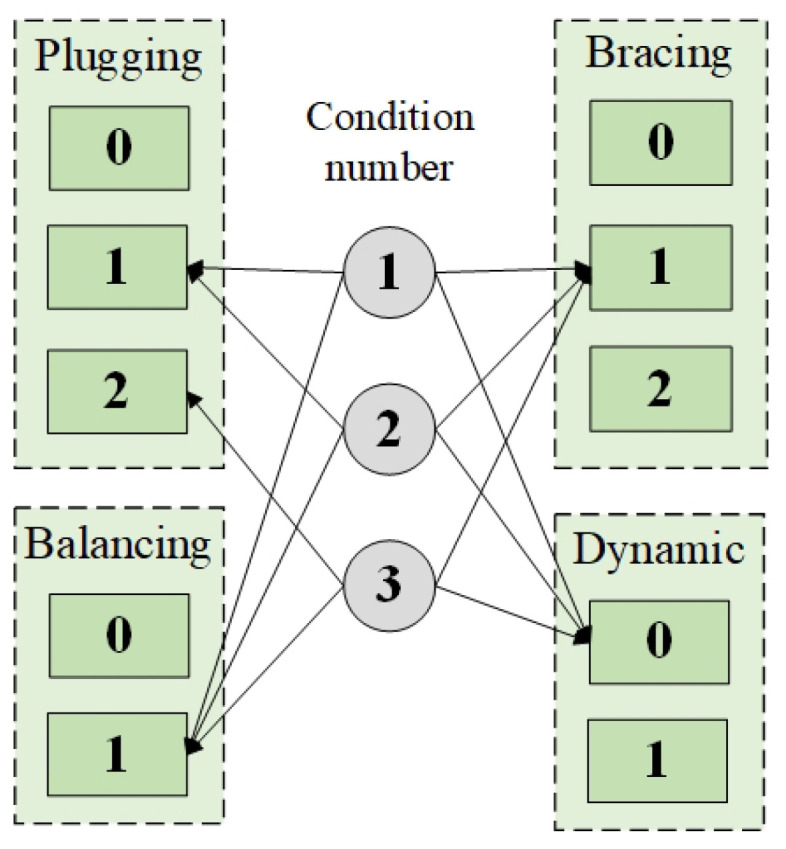
Auxiliary decision-making measures (Corresponding to Figure 7) under different working conditions (Table 4).

**Figure 17:**
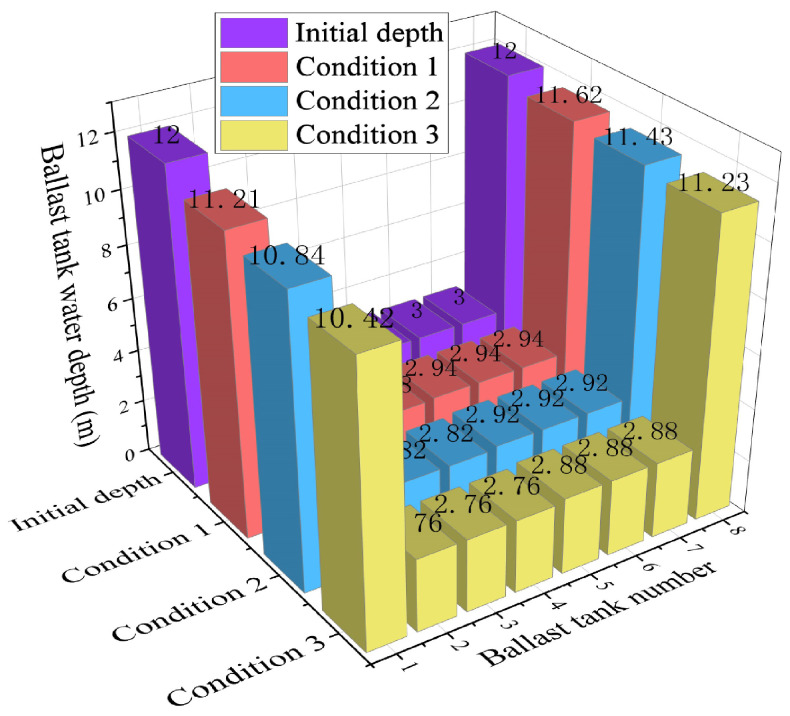
The variation in water depth for each ballast tank at different response times.

**Figure 18:**
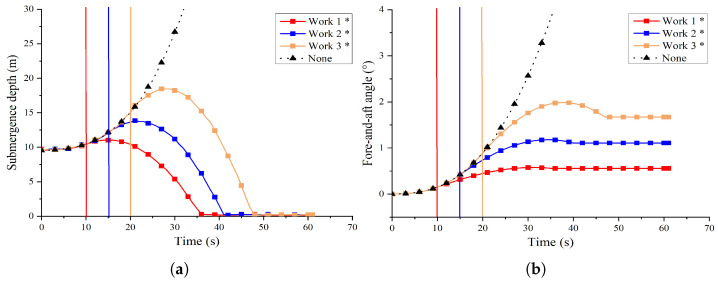
The state changes under different response times. (**a**) Submergence depth. (**b**) Fore-and-aft angle.

**Figure 19:**
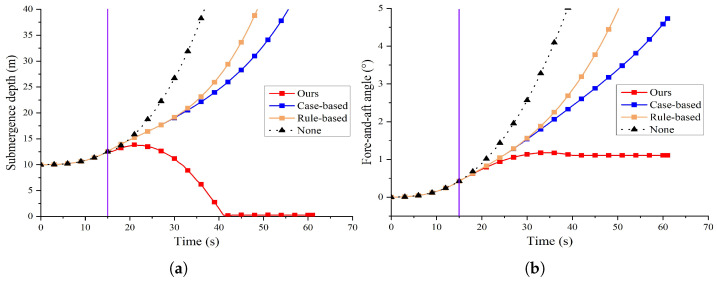
The state changes based on different methods (condition 2 from Table 4). (**a**) Submergence depth. (**b**) Fore-and-aft angle.

For the three working conditions with different break sizes, the strategy based on the ADMS is illustrated in Figure 16. At different response times, the change in water depth for each ballast tank under different working conditions is shown in Figure 17, while Table 5 summarizes the usage of HPG under different working conditions. The variation in the MUV state parameters at different response times (Table 4) is shown in Figure 18. The longitudinal lines of different colors represent the auxiliary decision-making measures corresponding to different response times under different working conditions. ‘None’ indicates the parameter change when no auxiliary decision-making measures are implemented. Taking condition 2 in Table 4 as an example, the state changes based on different methods are shown in Figure 19a,b.

As shown in Figure 17, taking the No. 1 ballast water tank as an example, the water depth of the ballast tank is 11.21 m in condition 1, 10.84 m in condition 2, and 10.42 m in condition 3. As shown in Figure 18a, works 1, 2 and 3 surfaced at 36 s, 42 s and 48 s, respectively. Furthermore, compared to the case where no action was taken, the depth of all working conditions decreased to varying degrees after action was taken within the response time. As shown in Figure 18b, after the measures were taken, work 3 stabilized at 1.6° in approximately 47 s, work 2 stabilized at 1.2° in approximately 40 s, and work 1 stabilized at 0.5° in approximately 35 s. Furthermore, the fore-and-aft angles under all three working conditions were reduced to varying degrees after the measures were taken. Therefore, the response time also consequently affects the implementation of the measures, especially the demand for HPG, ultimately impacting parameter changes. Generally, the longer the response time, the greater the fore-and-aft angle, and also the longer the MUV takes to surface.

As shown in Figure 19a, the depth obtained using the method proposed in this paper reaches the surface in about 41 s, while the case-based method reaches 25 m and the reasoning-based method reaches 28 m. Moreover, the depth obtained using the latter two methods continues to decrease over time. As shown in Figure 19b, the angle obtained using the method proposed in this paper reaches at 1° in about 40 s, while the case-based method reaches 2.3° and the reasoning-based method reaches 2.8°. Moreover, the angle obtained using the latter two methods continues to increase gradually over time. It can be seen that the method proposed in this paper significantly improves upon reasoning-based and case-based methods in terms of both the depth and pitch angle results.

### 4.2. The Definition and Analysis of the Evaluation Criteria

In this paper, the rationality of the ADMS based on DRL is assessed by setting evaluation criteria. The evaluation criteria for the ADMS under the condition of a small submergence depth are set refer to the (successful disposal) safety standard (Equation (13)) [38].

(13) $$\;\left\{\begin{array}{c} D\left(t\right)=D_{0}+{\Delta d}_{max}\leq 30\\ \left|\theta_{max}\right|\leq 30^{\circ } \end{array}\right.$$

In Equation (13), $D\left(t\right)$ is the current submergence depth of the MUV; $D_{0}$ is the initial submergence depth; ${\Delta d}_{max}$ is the maximum depth offset of the MUV; and $\theta_{\max}$ is the maximum fore-and-aft angle of the MUV. In this paper, we assume that the limit-of-safety depth is 30 m, and the limit-of-safety angle is 30. The MUV buoyancy and stability vitality evaluation criteria are defined and analyzed, as presented in Equation (14).

(14) $$\;\left\{\begin{array}{c} I_{D}=\left(1-\frac{D\left(t\right)}{30}\right)\cdot 100\%\\ I_{\theta }=\left(1-\frac{\left|\theta_{max}\right|}{30}\right)\cdot 100\% \end{array}\right.$$

**Figure 20:**
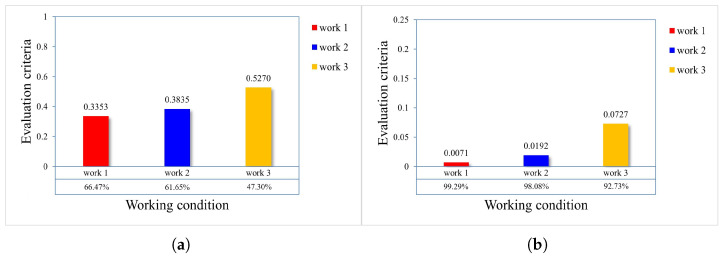
The histogram of the evaluation criteria in different breaks. (**a**) MUV buoyancy vitality. (**b**) MUV stability vitality.

**Figure 21:**
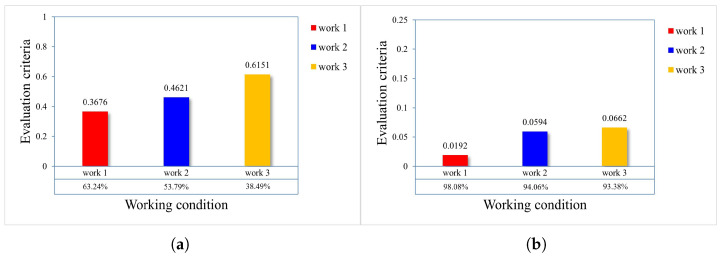
The histogram of the evaluation criteria at different response times. (**a**) MUV buoyancy vitality. (**b**) MUV stability vitality.

In Equation (14), $I_{D}$ is the MUV buoyancy vitality evaluation criterion; $I_{\theta }$ is the MUV stability vitality evaluation criterion. The evaluation criteria ($I_{D}$, $I_{\theta }$, Equation (14)) are analyzed based on the simulation results of the submergence depth and fore-and-aft angle (fore-and-aft angle takes absolute value). The histogram of the evaluation criteria in different breaks and at different response times is shown in Figure 20 and Figure 21. And all percentage results are calculated from the parameter results shown in Figure 14 and Figure 18 using Equation (14).

In the auxiliary decision-making process for the MUV in different breaks, the values of the MUV buoyancy vitality evaluation criterion are 66.47%, 61.65% and 47.30%. In the auxiliary decision-making process for the MUV at different response times, the values of the MUV buoyancy vitality evaluation criterion are 63.24%, 53.79% and 38.49%. Regarding the submergence depth parameter, all three working conditions fall within the safe range of 30 m, which is acceptable. The values of the MUV stability vitality evaluation criterion in different breaks are 99.29%, 98.08% and 92.73%. The values of the MUV stability vitality evaluation criterion at different response times are 98.08%, 94.06% and 93.38%. Regarding the fore-and-aft angle parameter, firstly, these absolute values are within the safe range of 30. Secondly, the closer to 0, the better. Therefore, the MUV buoyancy vitality evaluation criterion values are within the safe range (0–100%), and can exceed 38%. Meanwhile, the MUV stability vitality evaluation criterion values are also within the safe range, and can surpass 92%, with an optimal value exceeding 99%.

## 5. Conclusions

In this paper, an auxiliary decision-making system (ADMS) for manned underwater vehicle (MUV) damage management based on deep reinforcement learning (DRL) is proposed to provide decision support in emergencies and save the vitality of the MUV. In the proposed ADMS, a comprehensive States–Actions cluster encompassing various damage management measures for real MUV damage scenarios is constructed and digitized. According to the vital parameters of the damage control auxiliary decision-making, several novel reward functions are developed to ensure the DRL model obtains a safe strategy with ADMS operations for MUV damage management. The state–action value functions are developed based on function approximation techniques, which leverage experience replay and a uniform sampling mechanism to train the deep neural network model. This model is utilized as a nonlinear function approximator to determine the Q-values of the States–Actions cluster. The target network mechanism is used to update the parameters of the state–action value function based on a neural network. Finally, the optimal decision determined by the reinforcement learning (RL) decision model is provided, after which the state parameter results are computed using the environment model. The results of the simulation comparison and evaluation criteria show that the auxiliary decision-making measures provided by the ADMS under MUV damage scenarios are effective and rational. The following conclusions are obtained from the simulation results: By setting the evaluation criteria, the rationality of the ADMS based on DRL assesses whether the MUV is in a safe state. The MUV buoyancy vitality evaluation criterion values are within the safe range (0–100%) and can exceed 38%. Meanwhile, the MUV stability vitality evaluation criterion values are also within the safe range and can surpass 92%, with an optimal value exceeding 99%.

The proposed method in this paper is applicable to scenarios where a single-shell MUV experiences cabin flooding due to an equivalent breach caused by damage to the sea pipeline within a shallow diving depth range. In the future, physical models of the cabin and MUV can be established to verify the effectiveness of the proposed method. Using this model’s experimental data, deep learning (DL) methods for time-series processing can be applied to make predictions about the situation. In addition, this paper assumes the accuracy of state perception and situation prediction in the introduction; however, these two problems require further investigation in future studies.

## Figures and Tables

**Table 1:** Parameters of the neural network and algorithm.

Parameters	Values
Network layers	4
Number of neurons per layer	7/12/24/48
Learning rate	0.01
Discount factor	0.9
Greedy parameter	0.9
Number of training rounds	1000
Batch size	128

**Table 2:** Design of different break size conditions.

Parameters	Values
Condition number	1/2/3
Damaged cabin number	2
Crevasse radius (m)	0.1/0.5/1.0
Initial submergence depth (m)	10
Response time (s)	10

**Table 3:** Use of HPG in different breaks.

Condition Number	1	2	3
Initial HPG (%)	100	100	100
Residual HPG (%)	99.88	98.14	92.54

**Table 4:** Design of different response time conditions.

Parameters	Values
Condition number	1/2/3
Damaged cabin number	2
Crevasse radius (m)	0.5
Initial submergence depth (m)	10
Response time (s)	10/15/20

**Table 5:** Use of HPG at different response times.

Condition Number	1	2	3
Initial HPG (%)	100	100	100
Residual HPG (%)	98.14	97.26	93.29

## Data Availability

The datasets generated or analyzed during the current study are not publicly available without the consent of all authors but are available from the corresponding author on reasonable request.

## References

[1] Braidotti L., Prpic-Orsic J., Valcic M., Mauro F., Bucci V. The Ship Safety From Seafarers Perspective: Application of Fuzzy AHP for Decision Support. Proceedings of the 14th Baska GNSS Conference: Technologies, Techniques and Applications Across PNT and the 1st Workshop on Smart Blue and Green Maritime Technologies.

[2] Li Z., Yang D., Yin G. (2023). Ship Flooding Time Prediction Based on Composite Neural Network. J. Mar. Sci. Eng..

[3] Vychuzhanin V., Rudnichenko N., Boyko V., Shibaeva N., Konovalov S. (2016). Devising a Method for the Estimation and Prediction of Technical Condition of Ship Complex Systems. East. Eur. J. Enterp. Technol..

[4] Pennanen P., Ruponen P., Ramm-Schmidt H. Integrated decision support system for increased passenger ship safety. Proceedings of the Damaged Ship III.

[5] Vassalos D., Mujeeb A.M.P. (2021). Conception and Evolution of the Probabilistic Methods for Ship Damage Stability and Flooding Risk Assessment. J. Mar. Sci. Eng..

[6] Chang C.H., Kontovas C., Yu Q., Yang Z. (2021). Risk assessment of the operations of maritime autonomous surface ships. Reliab. Eng. Syst. Saf..

[7] Zhu L., Ren K., Pu J. Framework case decision reasoning method integrating multiple information. Proceedings of the Sixth International Conference on Electromechanical Control Technology and Transportation (ICECTT 2021).

[8] Li H., Guo J.Y., Yazdi M., Nedjati A., Adesina K.A. (2021). Supportive emergency decision-making model towards sustainable development with fuzzy expert system. Neural Comput. Appl..

[9] Calabrese F., Cataldo M., Corallo A., Pascalis A.D., Mancarella L., Ostuni L., Zizzari A.A. (2012). Damage Control System: An Application for Ship Safety and Security. IFAC Proc. Vol..

[10] Calabrese F., Corallo A., Margherita A., Zizzari A.A. (2012). A knowledge-based decision support system for shipboard damage control. Expert Syst. Appl..

[11] Hu L., Ma K., Ji Z. (2013). AM–H method-based decision support system for flooding emergencies onboard warship. Ocean Eng..

[12] Li A., Pu J.Y. (2013). Intelligent Decision Support System in Damage Control of Damaged Ship. Adv. Mater. Res..

[13] Park D., Shin Y., Chung J., Jung E.S. (2016). Development of damage control training scenarios of naval ships based on simplified vulnerability analysis results. Int. J. Nav. Archit. Ocean Eng..

[14] Kim S., Kang J., Choi B., Lee D., Kang H.J. (2020). RFID- and MEMS-based onboard crew location recognition system for incident response. J. Adv. Mar. Eng. Technol..

[15] Iqbal M. (2021). Rule-Based Reasoning for Decision Support in Search and Rescue. Master’s Thesis.

[16] Zhao X., Wang C., Cui P., Sun G. (2022). Operational Rule Extraction and Construction Based on Task Scenario Analysis. Information.

[17] Storvik M.H.R. (2020). Case-Based Reasoning for Decision Support in Search and Rescue. Master’s Thesis.

[18] Louvros P., Stefanidis F., Boulougouris E., Komianos A., Vassalos D. (2023). Machine Learning and Case-Based Reasoning for Real-Time Onboard Prediction of the Survivability of Ships. J. Mar. Sci. Eng..

[19] Lee D., Kim S., Lee K., Shin S., Choi J., Park B.J., Kang H.J. (2021). Performance-based on-board damage control system for ships. Ocean Eng..

[20] Bolbot V., Theotokatos G., Wennersberg L., Faivre J., Vassalos D., Boulougouris E., Rodseth O.J., Andersen P., Pauwelyn A.S., Coillie A.V. (2021). A novel risk assessment process: Application to an autonomous inland waterways ship. Proc. Inst. Mech. Eng. Part O J. Risk Reliab..

[21] Kiran B.R., Sobh I., Talpaert V., Mannion P., Al Sallab A.A., Yogamani S., Perez P. (2021). Deep reinforcement learning for autonomous driving: A survey. IEEE Trans. Intell. Transp. Syst..

[22] Li L., Wu D., Huang Y., Yuan Z.M. (2021). A path planning strategy unified with a COLREGS collision avoidance function based on deep reinforcement learning and artificial potential field. Appl. Ocean Res..

[23] Mohammadi R., He Q. (2022). A deep reinforcement learning approach for rail renewal and maintenance planning. Reliab. Eng. Syst. Saf..

[24] Yao J., Ge Z. (2022). Path-Tracking Control Strategy of Unmanned Vehicle Based on DDPG Algorithm. Sensors.

[25] Qi D., Zhao Y., Li L., Jia Z. (2024). Optimization of Predefined-Time Agent-Scheduling Strategy Based on PPO. Mathematics.

[26] Li Y., Li A., Ren K., Liu H., Jin T. (2023). Submarine Vitality.

[27] Cooke J.C.M. (1945). Handbook of Damage Control.

[28] Chen S.H., Ren Y.Q., Jiang L.J. (2016). Ship damage control technology. Ocean Eng. Equip. Technol..

[29] Watkins C.J.C.H., Dayan P. (1992). Q-learning. Mach. Learn..

[30] Jang B., Kim M., Harerimana G., Kim J.W. (2019). Q-Learning Algorithms: A Comprehensive Classification and Applications. IEEE Access.

[31] Heer B., MauBner A. (2024). Function Approximation. Dynamic General Equilibrium Modeling.

[32] Xu X., Zuo L., Huang Z.H. (2014). Reinforcement learning algorithms with function approximation: Recent advances and applications. Inf. Sci..

[33] Yang S.B., Ting T.O., Man K.L., Guan S.U. (2013). Investigation of Neural Networks for Function Approximation. Procedia Comput. Sci..

[34] Andrychowicz M., Denil M., Gomez S., Hoffman M.W., Pfau D., Schaul T., Shillingford B., de Freitas N. (2016). Learning to learn by gradient descent by gradient descent. arXiv.

[35] Jiang R., Zhang S., Chelu V., White A., van Hasselt H. Learning expected emphatic traces for deep RL. Proceedings of the 36th AAAI Conference on Artificial Intelligence.

[36] Lee D., Song J. (2023). Risk-informed operation and maintenance of complex lifeline systems using parallelized multi-agent deep Q-network. Reliab. Eng. Syst. Saf..

[37] Zhang Q., Liu Y., Xiang Y., Xiahou T. (2024). Reinforcement learning in reliability and maintenance optimization: A tutorial. Reliab. Eng. Syst. Saf..

[38] Hu K., Huang H.F., He B., Zhang P. (2021). Submarine large depth loss buoyancy simulation analysis and manipulation method. J. Ordnance Equip. Eng..

